# Joint CB1 and NGF Receptor Activation Suppresses TRPM8 Activation in Etoposide-Resistant Retinoblastoma Cells

**DOI:** 10.3390/ijms25031733

**Published:** 2024-01-31

**Authors:** Szymon Ludwiczak, Jacqueline Reinhard, Peter S. Reinach, Aruna Li, Jakub Oronowicz, Aisha Yousf, Vinodh Kakkassery, Stefan Mergler

**Affiliations:** 1 Department of Ophthalmology, Charité—Universitätsmedizin Berlin, Corporate Member of Freie Universität Berlin and Humboldt-Universität zu Berlin, 10117 Berlin, Germany; szymon.ludwiczak@charite.de (S.L.); aruna.li@charite.de (A.L.); 2 Department of Cell Morphology and Molecular Neurobiology, Faculty of Biology and Biotechnology, Ruhr University Bochum, 44801 Bochum, Germany; jacqueline.reinhard@rub.de (J.R.); aisha.yousf@rub.de (A.Y.); 3 School of Ophthalmology and Optometry, Wenzhou Medical University, Wenzhou 325015, China; preinach25@gmail.com; 4 Malteser Waldkrankenhaus Clinic for Orthopedics and Trauma Surgery, 91054 Erlangen, Germany; jakub.oronowicz@waldkrankenhaus.de; 5 Department of Ophthalmology, Clinic Chemnitz, 09116 Chemnitz, Germany; 6 Department of Ophthalmology, University of Luebeck, 23538 Luebeck, Germany

**Keywords:** retinoblastoma, chemotherapy resistance, transient receptor potential channel vanilloid 1, transient receptor potential channel melastatin 8, etoposide, nerve growth factor, cannabinoid receptor 1, Ca^2+^ signaling

## Abstract

In childhood, retinoblastoma (RB) is the most common primary tumor in the eye. Long term therapeutic management with etoposide of this life-threatening condition may have diminishing effectiveness since RB cells can develop cytostatic resistance to this drug. To determine whether changes in receptor-mediated control of Ca^2+^ signaling are associated with resistance development, fluorescence calcium imaging, semi-quantitative RT-qPCR analyses, and trypan blue dye exclusion staining patterns are compared in WERI-ETOR (etoposide-insensitive) and WERI-Rb1 (etoposide-sensitive) cells. The cannabinoid receptor agonist 1 (CNR1) WIN55,212-2 (40 µM), or the transient receptor potential melastatin 8 (TRPM8) agonist icilin (40 µM) elicit similar large Ca^2+^ transients in both cell line types. On the other hand, NGF (100 ng/mL) induces larger rises in WERI-ETOR cells than in WERI-Rb1 cells, and its lethality is larger in WERI-Rb1 cells than in WERI-ETOR cells. NGF and WIN55,212-2 induced additive Ca^2+^ transients in both cell types. However, following pretreatment with both NGF and WIN55,212-2, *TRPM8* gene expression declines and icilin-induced Ca^2+^ transients are completely blocked only in WERI-ETOR cells. Furthermore, *CNR1* gene expression levels are larger in WERI-ETOR cells than those in WERI-Rb1 cells. Therefore, the development of etoposide insensitivity may be associated with rises in *CNR1* gene expression, which in turn suppress *TRPM8* gene expression through crosstalk.

## 1. Introduction

Retinoblastoma (RB) was described as early as 1597 by Pieter Pawius [1]. It can be a life-threatening condition that is the most common primary intraocular tumor in childhood [2,3]. This disease arises from malignant cells that form in the tissues of the retina. Therapeutic management can have variable success due to differences in sensitivity to etoposide, which affects cell survival and tumor progression. However, the mechanisms that account for variations in etoposide sensitivity and long-term survival are not well understood. Despite this limitation, novel procedures are now employed which improve survival [4,5,6]. In Europe and in the US, advanced diagnosis and therapy have reduced mortality to 5% [7]. However, in less developed countries the survival rates remain lower because they have limited access to this novel lifesaving technology. Therefore, novel procedures are still needed to improve therapeutic management of RB-induced cancer progression.

The transient receptor potential melastatin 8 (TRPM8) channel is also known as the cold or menthol receptor, and it is a member of the TRP superfamily. Even though there is no TRPM8 expression in the retina, RB cells express such activity [8,9,10]. Therefore, TRPM8 is suggested as a molecular biomarker and therapeutic target for developing selective cancer treatment, since this channel is functional in etoposide-sensitive cancerous tumor types [11,12]. For example, TRPM8 channels regulate neurotensin secretion in neuroendocrine tumor cells [13]). In addition, TRPM8 is involved in calcium regulation in ocular tumors, such as uveal melanoma [14] and RB cells [8,9,15]. The commonality between TRPM8 expression modulation and progression of many diverse types of cancer accounts for the increasing efforts to assess the effectiveness of using TRPM8 as a drug target for cancer therapy [12].

Cannabinoids are used to treat many diseases [16]. Cannabinoid receptor subtypes 1 (CB1) and CB 2 (CB2) are widely expressed in the human endocrine, gastrointestinal, immune, and nervous systems [17,18,19]. CB1 receptors are G protein-coupled receptors (GPCRs) [19] and they mediate control of TRP subtype activity through crosstalk (GPCR-TRP axis) [20]. Cannabinoids have been proposed for use in cancer therapeutics [21,22,23]. Compared to CB2, CB1 is mainly localized in the human anterior eye and retina, as well as in the trabecular meshwork, and their different expressions in combination with TRPV channels imply a potential role of these CB targets in primary and recurrent pterygia [24,25,26,27]. Notably, functional CB1 expression interacts with TRPs through crosstalk in RB cells [8].

Nerve growth factor (NGF) is a neurotrophic factor whose levels are almost four times higher in patients with RB compared to the control group [28]. NGF is, therefore, thought to be involved in tumor signaling pathways, as RB cells mediate a larger response to neurotrophins [29]. RB cells that are sensitive to chemotherapy express TrkA, which is an NGF receptor for NGF [30]. NGF deficiency may trigger spontaneous regression of chemotherapeutic-sensitive RB cells, whereas aggressive RB cells have defective apoptotic machinery, which enables them to evade apoptosis and confers resistance to etoposide treatment [31]. Furthermore, there is also a close relationship in other tissues between tropomyosin receptor kinase A (TrkA) and other TRP channel subtypes [32] and CB1 [33].

*TRPA1* gene expression is downregulated in etoposide-resistant WERI-ETOR cells relative to its expression in the etoposide-sensitive WERI-Rb1 counterpart whereas *TRPV1*, *TRPM8*, and *CB1* gene expression is present in both cell lines [8,9]. Notably, the capsaicin- and heat-induced Ca^2+^ increase caused by TRPV1 activation is prompt in the WERI-ETOR etoposide-resistant cells but somewhat delayed in the WERI-Rb1 etoposide-sensitive counterpart [8].

In the current study, we determined if Ca^2+^ signaling mediates crosstalk between CB1, TRP, and NGF receptors, since such an interaction may modulate RB cell viability and survival. Accordingly, their individual and combined roles in modulating RB cell survival are evaluated based on determining if there is an association between the effects of CB1 activation on *NGF* and *TRPM8* gene expression levels, induced Ca^2+^ signaling, and RB cell survival.

## 2. Results

### 2.1. NGF Upregulates CB1 Gene Expression in WERI-ETOR Cells

**Figure 1:**
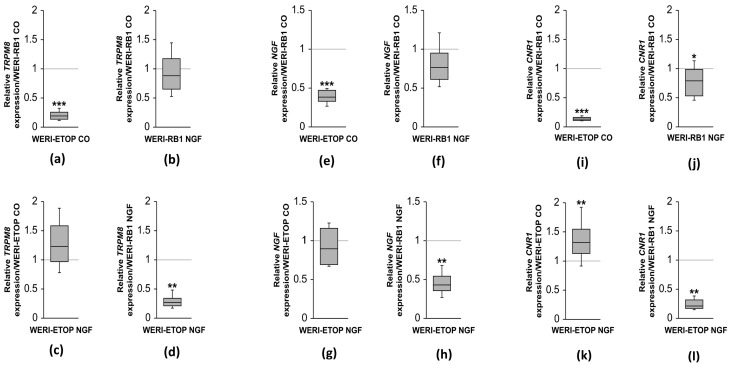
Relative mRNA expression levels of *TRPM8*, *NGF*, and *CB1* (*CNR1*) in WERI-ETOR and WERI-Rb1 cells with NGF (100 ng/mL) and without NGF treatment (CO = control; *n* = 5–6, * *p* < 0.05, ** *p* < 0.01, *** *p* < 0.001). (**a**–**d**) The *TRPM8* mRNA level is significantly downregulated in WERI-ETOR cells compared to WERI-Rb1 cells, irrespective of NGF treatment. (**e**–**h**) *NGF* elicits the same expression pattern. (**i**–**l**) Also, *CNR1* is significantly lower expressed in WERI-ETOR cells compared to WERI-Rb1 cells. Notably, NGF treatment leads to a significant upregulation of the *CNR1* expression level in WERI-ETOR cells.

NGF is a classical activator of the MAPK signaling pathway in RB cells [34]. We performed RT-qPCR analyses to determine if this signaling pathway includes changes in *TRPM8*, *NGF*, and *CNR1* mRNA expression patterns that are different in etoposide-sensitive WERI-Rb1 than those in etoposide-resistant WERI-ETOR cells. Figure 1 shows the individual effects of *NGF* on the *TRPM8*, *NGF*, and *CNR1* expression levels in these cell lines. The results are separated into different panel pairs for the two cell types in which the mRNA expression level under control conditions (CO, control) was compared to the level in the presence of NGF (100 ng/mL), i.e., (1) *TRPM8* a–d; (2), *NGF* e–h, and (3) *CB1* (*CNR1*) i–l in Figure 1. The *TRPM8* mRNA level was downregulated in WERI-ETOR cells compared to WERI-Rb1 cells irrespective of NGF treatment (CO: 0.192-fold; *** *p* < 0.001; NGF: 0.269-fold; ** *p* = 0.001; Figure 1a,d). Similar *TRPM8* mRNA expression levels were evident in NGF treated cells compared to the respective control group in both cell lines (WERI-Rb1: 0.878-fold; *p* = 0.328; WERI-ETOR: 1.229-fold; *p* = 0.088, Figure 1b,c). Figure 1e–h shows the NGF mRNA expression levels in both cell lines with and without NGF treatment. (Figure 1e–h). The *NGF* mRNA expression level was significantly lower in WERI-ETOR cells than in WERI-Rb1 cells with or without NGF treatment (CO: 0.384-fold; *** *p* < 0.001; NGF: 0.426-fold; ** *p* < 0.01; Figure 1e,h). On the other hand, *NGF* mRNA expression levels were similar in non-treated and NGF-treated WERI-Rb1 cells and WERI-ETOR cells (WERI-Rb1 NGF: 0.762-fold; *p* = 0.065; WERI-ETOR NGF: 0.893-fold; *p* = 0.21; Figure 1f,g).

*CNR1* mRNA expression levels are significantly reduced in NGF and non-treated WERI-ETOR cells compared to those in WERI-Rb1 cells (CO: 0.132-fold, *** *p* < 0.001; NGF: 0.212-fold; ** *p* < 0.01; Figure 1i,l). However, the *CNR1* expression level was significantly upregulated in NGF-treated WERI-ETOR cells compared to that in non-treated WERI-ETOR cells (1.32-fold; ** *p* < 0.01; Figure 1k). On the other hand, the *CNR1* mRNA level was significantly downregulated in NGF-treated WERI-Rb1 cells compared to non-treated WERI-Rb1 cells (0.789-fold; * *p* = 0.03; Figure 1j). Overall, *TRPM8*, *NGF*, and *CNR1* gene expression levels were significantly lower in WERI-ETOR cells than in WERI-Rb1 cells irrespective of the NGF treatment. Interestingly, *CNR1* mRNA expression was significantly upregulated in the NGF-treated WERI-ETOR cells compared to that in non-treated WERI-ETOR cells.

### 2.2. Analysis of Cell Viability of WERI-Rb1 Cells

**Figure 2:**
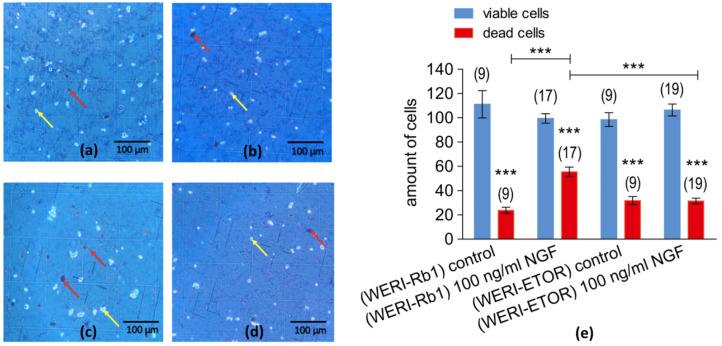
Microscopic images of trypan blue staining of RB cells. The microscopic images (scale bar 100 µm) show dead cells (dark dots), which were marked with red arrows and the living cells (light dots) with yellow arrows. (**a**) WERI-RB1 cells after 5 days of cell cultivation without NGF application (control group). (**b**) WERI-ETOR after 5 days of cell cultivation without NGF application (control group). (**c**) Same group as shown in (**a**) but after 5 days of incubation with 100 ng/mL NGF. (**d**) Same group as shown in (**b**) but after 5 days of incubation with 100 ng/mL NGF. (**e**) Comparison of NGF-induced lethality in in WERI-Rb1 and in WERI-ETOR cells. The numbers in brackets indicate the number of measurements. The number of viable (in blue) and dead cells (in red) of etoposide-sensitive (WERI-Rb1) and etoposide-resistant (WERI-ETOP) is shown in the Y-axis. RB cell cultures with and without NGF treatment. Cells are incubated with 100 ng/mL NGF, and cell number is determined after 5 days. Untreated cells (without NGF) served as controls. Declines in cell viability are larger in NGF-treated WERI-Rb1 cells than in WERI-ETOR cells. NGF enhances etoposide-induced cytotoxicity more in WERI-Rb1 cells than in WERI-ETOR cells. *** *p* < 0.001; were considered statistical significance between groups.

To determine the effect of 100 ng/mL NGF for 5 days on cell death, the trypan blue dye exclusion assay evaluated changes in RB cell density. Figure 2a–d shows typical microscopic images containing dead and living WERI-Rb1 cells as well as WERI-ETOR cells. The living cells are regularly in excess and approximately equally distributed (light dots under the conditions shown in Figure 2a–d). After 5 days of exposure to 100 ng/mL NGF, NGF increased the proportion of dead WERI-Rb1 cells (Figure 2c).

Figure 2e compares the number of dead and live cells in WERI-Rb1 and WERI-ETOR cells. A significantly larger number of dead cells were present in NGF-treated WERI-Rb1 cells (45 ± 4; *n* = 17) than in the untreated WERI-Rb1 cells (24 ± 3; *n* = 9; *** *p* < 0.001) (Figure 2e).

### 2.3. Comparison of Blunting Effects of AMTB on TRPM8 Activation in WERI-Rb1 and WERI-ETOR Cells

**Figure 3:**
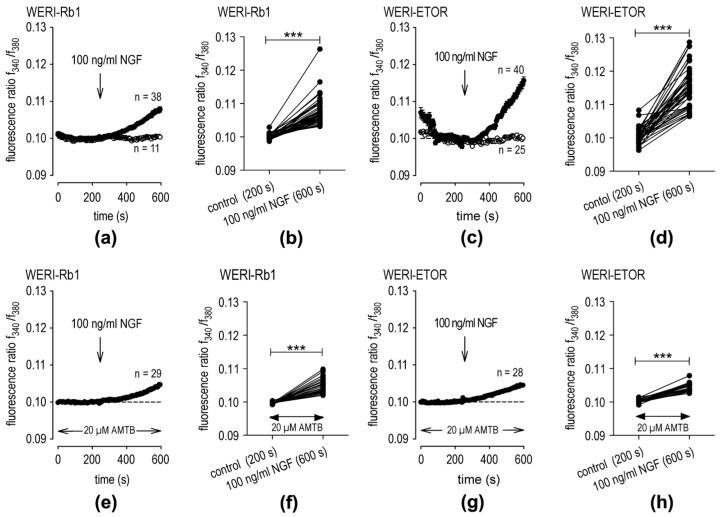
AMTB blunts NGF-induced increases in intracellular Ca^2+^. (**a**) The time-dependent changes are shown as relative intracellular Ca^2+^ levels in fura-2/AM-loaded WERI-Rb1 cells. Data are shown as mean ± SEM. n indicates the number of cells examined in this set of experiments. The dashed line shows the baseline value (0.1). Arrows indicate the application of NGF (100 ng/mL). NGF induces an increase in Ca^2+^ influx in WERI-Rb1 cells (*n* = 38; filled circles), whereas the fluorescence ratio is invariant in the control cells (*n* = 11; open circles). (**b**) Single data at 200 s (control) versus 600 s. NGF increases the fluorescence ratio in each measurement (*n* = 38; *** *p* < 0.001; paired tested). (**c**) Same experiment as explained in panel (**a**) but with WERI-ETOR cells (filled circles; *n* = 40) and a control base line (open circles; *n* = 25). The NGF-induced increase in the f340 nm/f380 nm fluorescence ratio was at higher levels compared to the WERI-Rb1 cells (*n* = 38–40; *** *p* < 0.001; paired tested). (**d**) Same diagram as shown in panel (**b**) but with WERI-ETOR cells also showing an increase in the fluorescence by NGF (*n* = 40; *** *p* < 0.001; paired tested), but at higher levels compared to the WERI-Rb1 cells (*n* = 38–40; *** *p* < 0.001; unpaired tested). (**e**–**h**) Same experiments as shown in panels (**a**–**d**) but in the presence of 20 µM AMTB (*n* = 28–29).

The effects of NGF-induced rises in TRPM8 activity in the two different cell lines are compared. This assessment entails comparing the inhibitory effects of TRPM8 blocker AMTB [35,36] on rises in TRPM8 activity. In the WERI-Rb1 cells, NGF (100 ng/mL) increased the f340 nm/f380 nm fluorescence ratio from 0.09998 ± 0. 0.00012 at t = 200 s (*n* = 38) to 0.10780 ± 0.00070 at t = 600 s (*n* = 38; *** *p* < 0.001; paired tested) (Figure 3a,b). The rise that occurred in WERI-ETOR cells was slightly higher (*n* = 38–40; *p* < 0.001; unpaired tested). In these cells, NGF increased the fluorescence ratio from 0.10050 ± 0.00039 at t = 200 s (*n* = 40) to 0.11520 ± 0.00096 at t = 600 s (*n* = 40; *** *p* < 0.001; paired tested) (Figure 3c,d).

Notably, this effect could be suppressed at the same level by AMTB in both cell lines (WERI-Rb1: 0.1047 ± 0.0004 at t = 600 s (*n* = 29); WERI-ETOR cells: 0.1045 ± 0.0002 (*n* = 28; *p* > 0.05; unpaired tested) (Figure 3e–h). Taken together, NGF led to a significantly larger rise in intracellular Ca^2+^ in WERI-ETOR cells than in the WERI-Rb1 cells, and AMTB partially suppressed this effect to the same level in both cell lines.

### 2.4. NGF- and WIN55,212-2-Induced TRPM8 Activation Only Occurs in WERI-ETOR Cells

**Figure 4:**
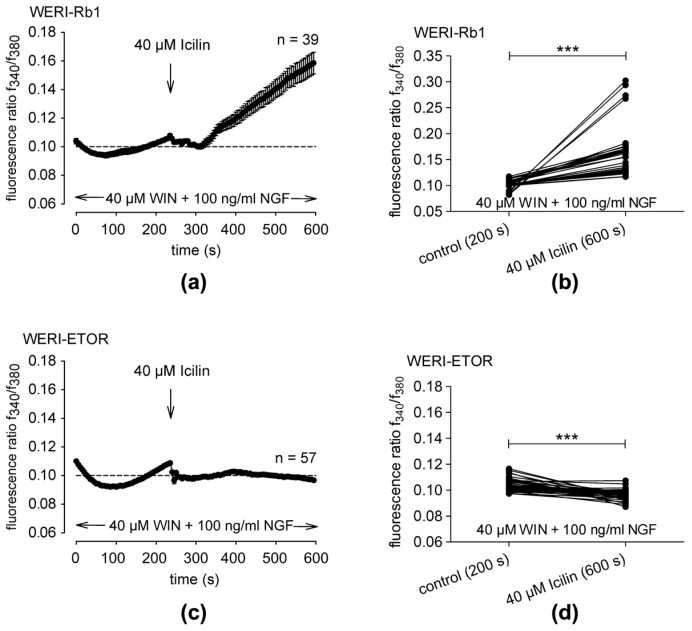
Crosstalk mediating NGF and CB1-induced TRPM8 activation: (**a**) Fluorescence ratio data ta are represented as mean ± SEM. n indicates the number of cells in this set of experiments. The dashed line represents the baseline value (0.1). Arrows indicate the application of icilin (40 µM) in the presence of 40 µM WIN55,212-2 and 100 ng/mL NGF. Icilin induces an increase in Ca^2+^ influx in WERI-Rb1 cells (*n* = 39; filled circles). (**b**) Single data at 200 s (control) versus 600 s (icilin in the presence of WIN55,212-2 and NGF) show that the f340 nm/f380 nm fluorescence ratio only rises in WERI-Rb1 cells (*n* = 39; *** *p* < 0.001). (**c**) Same experiment as explained in panel (**a**) but with WERI-ETOR cells (filled circles; *n* = 57). The icilin-induced rise is absent in WERI-ETOR cells. (**d**) Same conditions as those in panel (**b**); unlike with WERI-Rb1 cells, icilin fails to induce rises in intracellular Ca^2+^ that are induced under the same conditions in WERI-ETOR cells.

In the combined presence of the CB1 agonist WIN55,212-2 (40 µM) and NGF (100 ng/mL), icilin induced a robust increase in the fluorescence ratio from 0.10230 ± 0.00115 at t = 200 s (*n* = 39) to 0.15850 ± 0.00762 at t = 600 s (*n* = 39; *** *p* < 0.001; paired tested) in the WERI-Rb1 cells (Figure 4a,b). In contrast, this response to icilin was completely abolished in WERI-ETOR cells. Specifically, icilin (40 µM) instead even slightly decreased the fluorescence ratio from 0.10330 ± 0.00060 at t = 200 s (*n* = 39) to 0.09666 ± 0.0004763 at t = 600 s (*n* = 57; *** *p* < 0.001; paired tested) in WERI-ETOR cells (Figure 4c,d). Taken together, crosstalk between CB1 and NGF activation of TRPM8 only occurs in WERI-Rb1 cells.

### 2.5. CB1 and NGF Mediates TRPM8 Activation through Crosstalk Only in WERI-ETOR

**Figure 5:**
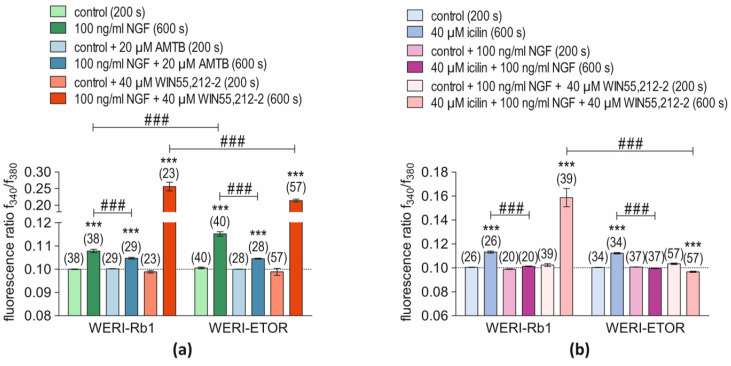
Comparison of the effects of NGF, AMTB, icilin, and WIN55,212-2 on Ca^2+^ signaling responses in WERI-Rb1 and WERI-ETOR cells. The numbers in brackets indicate the number of cells. The respective control groups are marked with congruent lighter shades of color. (**a**) 100 ng/mL NGF (green bar) increased the fluorescence ratios in both cell lines, but in WERI-ETOR cells these rises stabilize at higher levels (*n* = 38–40; ^###^ *p* < 0.001) than in WERI-Rb1 cells, which 20 µM AMTB suppresses in both cell lines (*n* = 28–29; ^###^ *p* < 0.01) (blue bars). WIN55,212-2 (40 µM) induces a diminished response in both cell lines in WERI-ETOR (*n* = 23–57; ^###^ *p* < 0.001) (red bars). (**b**) The individual effects are described of icilin on these different responses are shown in panel A, where 40 µM icilin increased the fluorescence ratio in both cell lines (*n* = 26–34; bright blue bars), which 100 ng/mL NGF suppressed (*n* = 20–37; both ^###^ *p* < 0.01, violet bars). With NGF and WIN55,212-2, the icilin-induced Ca^2+^ influx is only visible in WERI-Rb1 cells but is absent in WERI-ETOR cells (*n* = 39–57; ^###^ *p* < 0.01; pink bars). *** *p* < 0.001; were considered statistical significance between groups.

The possibility was evaluated that NGF and CB1 activation induce rises in TRPM8 activity through crosstalk. Accordingly, the effects of NGF on the fluorescence ratio in the presence of either AMTB or WIN55,212-2 or the effect of their combined presence were determined (NGF-group) (Figure 5a). Furthermore, the effects of icilin on the fluorescence ratio in the presence of either NGF or WIN55,212-2 or the effect of their combined presence are shown in Figure 5b (icilin group). An initial statistical analysis revealed that blockage of TRPM8 activation with AMTB (20 µM) partially suppressed the NGF-induced rise in Ca^2+^ influx in both cell lines to the same level (*n* = 28–29; *p* > 0.05) (Figure 5a). There were no significant differences between the rises in intracellular Ca^2+^ level induced by the shown conditions in the two different cell lines. NGF fully blocked the icilin-induced Ca^2+^ increase in both cell lines (Figure 5b). In WERI-Rb1 cells, the fluorescence ratio without NGF at 600 s was 0.1132 ± 0.00091 (*n* = 26), whereas NGF decreased it to 0.1013 ± 0.00029 (*n* = 20; ^###^ *p* < 0.001; unpaired tested). Similarly, NGF suppressed the ratio rise from 0.1122 ± 0.00058 (*n* = 34) to 0.09955 ± 0.00016 (*n* = 37; ^###^ *p* < 0.001; unpaired tested) (Figure 5b) in WERI-ETOR cells. On the other hand, WIN55,212-2 (40 µM) preincubation reversed the effect of NGF on the fluorescence ratio, since WIN55,212-2 induced a huge rise in both cell lines (Figure S1). Despite this large rise in both cell types, it was lower in WERI-ETOR (f340/f380 = 0.2141 ± 0.004090; *n* = 57) than in WERI-Rb1 cells. (f340/f380 = 0.2562 ± 0.013030; *n* = 23; ^###^ *p* < 0.001; unpaired tested) (Figure 5a). Without NGF, WIN55,212-2 increased the fluorescence ratio in both cell lines to the same level, which is higher than that of NGF (WERI-Rb1: f340/f380 = 0.13340 ± 0.00186; *n* = 54; *** *p* < 0.001; WERI-ETOR: f340/f380 = 0.13220 ± 0.00237; *n* = 61; *** *p* < 0.001, all paired tested, Figure S1). A similar effect occurred with icilin, which was smaller than that of either NGF or WIN55,212-2. The most significant difference was that in the presence of NGF and WIN55,212-2, icilin failed to induce a rise in the fluorescence ratio in WERI-ETOR cells. This difference in sensitivity to icilin exposure is evident, since icilin induced a rise in the fluorescence ratio in WERI-Rb1 cells (WERI-Rb1: f340/f380 = 0.1585 ± 0.007618; *n* = 39; WERI-ETOR: f340/f380 = 0.09666 ± 0.000476; *n* = 57; ^###^ *p* < 0.001; unpaired tested, Figure 4 and Figure 5b).

## 3. Discussion

### 3.1. Main Results

This study focuses on determining if differences in etoposide sensitivity in RB cells are associated with changes in crosstalk-mediated control by NGF and CNR1 of TRPM8 activation in etoposide-insensitive WERI-ETOR and etoposide-sensitive WERI-Rb1 cells. The TRPM8 and CNR1 levels of gene expression and magnitudes of Ca^2+^ transients are both smaller in WERI-ETOR cells than in WERI-Rb1 cells irrespective of NGF-treatment. In addition, CNR1 mRNA expression is upregulated in the NGF-treated WERI-ETOR cells. NGF-induced lethality is higher in WERI-Rb1 cells than in WERI-ETOR cells (Figure 2). Furthermore, the NGF-induced Ca^2+^ transients are larger in WERI-ETOR than those in the WERI-Rb1 cells and inhibition of TRPM8 activation by AMTB partially blunts NGF-induced rises in intracellular Ca^2+^ influx in both cell lines. Sensitivity of RB cells to etoposide lethality is dependent on the ability of NGF and CB1 to mediate icilin-induced activation of TRPM8. Icilin only induces a Ca^2+^ increase in the presence of NGF and WIN55,212-2 in WERI-Rb1 cells, whereas this rise is completely suppressed in WERI-ETOR cells (Figure 4 and Figure 5b). Moreover, NGF augmented a large WIN55,212-induced increase in Ca^2+^ influx. Their combined effect is larger in WERI-Rb1 cells than in WERI-ETOR cells (Figure 5a). The absence of TRPM8 responsiveness to icilin in the presence of NGF and WIN55,212 in WERI-ETOR cells suggests that TRPM8 upregulation may be a therapeutic strategy to mitigate declines in etoposide sensitivity.

### 3.2. Roles of TRPM8, NGF, and CB1 Gene Expression in WERI-Rb1 and WERI-ETOR Cells

*TRPM8* expression was verified based on its previous description in different isolated RB tissues [8]. The *TRPM8* gene expression level may be a factor that affects RB cell sensitivity to etoposide since its expression is lower in etoposide-insensitive WERI-ETOR cells than in the etoposide-sensitive counterpart [8,9]. Another observation supporting an association between TRPM8 functionality and etoposide sensitivity is that in other cytostatic-sensitive tumor cells, *TRPM8* remains overexpressed in prostate cancer cells [37]. Furthermore, increases in *TRPM8* gene expression are associated with rises in etoposide sensitivity during tumor progression in other studies. On the other hand, a lower *TRPM8* gene expression is generally a characteristic feature of cytostatic-resistant cells, such as WERI-ETOR cells, in contrast to WERI-Rb1 cells where *TRPM8* PCR signals are more evident [8]. Consequently, TRPM8 activation is proposed to act as a potential antitumor target, as reviewed in the literature [37].

In a comparative mRNA distribution profile of the human TRPM channel family, *TRPM8* mRNA expression level is higher in the prostate and liver [38]. Notably, an increase in *TRPM8* expression was observed in neuroendocrine tumor cells, and this rise was associated with an increased release of neurotensin [13]. This association parallels findings in WERI-Rb1 cells, where the *TRPM8* mRNA expression was clearly detectable, in contrast to various RB tissues of unknown etoposide sensitivity, where it was less detectable [8]. Moreover, in WERI-ETOR cells, both *NGF* and *TRPM8* expression are downregulated, even after NGF treatment (Figure 1d,h). This suggests potential alterations in NGF signaling in WERI-ETOR cells, considering that the pathogenesis of human proliferative diseases, such as prostate cancer, is similarly mediated by NGF signaling [39]. However, the signaling pathways whereby NGF and CNR1 modulate TRPM8 function and control of etoposide sensitivity require future clarification.

### 3.3. Roles of NGF in Controlling Cell Viability and Calcium Regulation

NGF-treatment of the etoposide-sensitive (i.e., normal) WERI-Rb1 cells is associated with increases in RB lethality whereas this is not the case in the etoposide-resistant cells. This difference helps to explain why NGF treatment is less cytotoxic in etoposide-resistant cells. NGF plays a crucial role in cell survival and apoptosis in neuronal cells due to its binding to tropomyosin receptor kinase A (TrkA) and to 75-kDa NGF receptor (NGFR) (p75^NTR^) [40]. Wu et al. compared growth rates in two different tumor cell lines. They found that a high growth rate is correlated with a low apoptotic rate and a high resistance to anoikis [41]. The lower *NGF* gene expression in WERI-ETOR than in the WERI-Rb1 cells (Figure 1e) may be associated with a higher apoptotic rate when the WERI-Rb1 cells are treated with NGF compared to non-treated controls (Figure 2a–d). In contrast, there was a lower apoptotic rate regardless of whether or not the WERI-ETOR cells were treated with NGF (Figure 2e). However, this finding contradicts with the higher levels of intracellular Ca^2+^ in WERI-ETOR cells following supplementation with NGF even though excessive Ca^2+^ influx can lead to apoptosis (calcium–apoptosis link) [42] (Figure 3). Higher levels of intracellular Ca^2+^ cells could also be registered after the addition of Asc in WERI-ETOR cells compared to WERI-Rb1 cells [9]. However, treatment of both cell lines with Asc decreased the cell density of both cell lines corresponding to increased lethality. This was not the case with NGF in WERI-ETOR cells in this study with a lower lethality (Figure 2e), indicating that NGF has a different mode of action than Asc. Regarding the NGF phenomenon, the complexity may be due to the *CB1* gene expression patterns shown in Figure 1, as they are different compared to those of *TRPM8* and *NGF*. Whereas the *CB1* mRNA level is slightly downregulated in NGF-treated WERI-Rb1 cells, the *CB1* mRNA expression level was upregulated in WERI-ETOR cells (Figure 1j,k). Since NGF is associated with TRPV1-induced Ca^2+^ influx and CB1 activation increases intracellular calcium via TRPV1 [32,33,43], one possible explanation may be that there is a link between NGF and the CB1 receptor-mediated Ca^2+^ influx through TRPM8. This Ca^2+^ influx is at higher levels in WERI-ETOR cells compared to WERI-Rb1 cells after addition of NGF (Figure 3).

### 3.4. Mechanism of Interaction between NGF and the CB1-TRPM8 Axis

**Figure 6:**
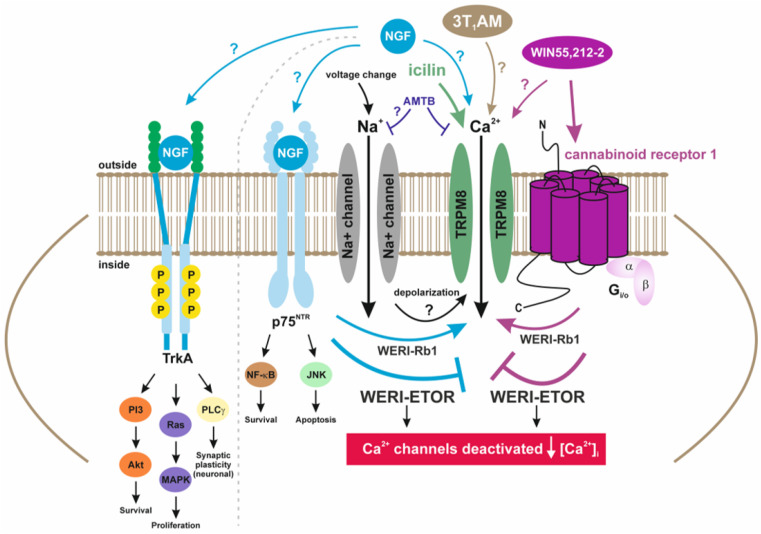
Hypothetical model that accounts for how interactions between NGF, CB1, and TRPM8 modulate rises in Ca^2+^ influx and the development of etoposide sensitivity. This hypothetical scheme proposes how CB1 and TrkA/p75^NRT^ receptor-linked signaling pathways induce increases in Ca^2+^ influx by activating TRPM8 in etoposide-sensitive WERI-Rb1 cells whereas TRPM8-induced Ca^2+^ influx is blocked in WERI-ETOR cells (Figure 5b, pink bars). The gray dashed line highlights the different signaling pathways between TrkA and p75^NRT^. Ca^2+^ channels, such as TRPs of the TRPM8 subtype (menthol receptor), can be selectively activated by moderate cooling (<≈25–28 °C) [51] or pharmacologically by icilin [52] or by thyronamines, such as 3T_1_AM [49], and blocked by AMTB [48], which may also suppress Na^+^ channels in RB cells [46]. NGF binds to the tropomyosin receptor kinase A (TrkA) with high affinity [53] and to p75 neurotrophin receptor (p75^NTR^) with low affinity. P75^NTR^ is linked to downstream pathways that modulate the balance between neuronal survival and neuronal death. When WIN55,212-2 activates CB1 in WERI-Rb1 cells, icilin-induced TRPM8 activation leads to an increase in intracellular Ca^2+^ (↑[Ca^2+^]_i_]) (Figure 4a). Notably, the opposite is the case in WERI-ETOR cells (↓[Ca^2+^]_i_]) (Figure 4c).

Even though RB cells are non-excitable tumor cells, it is still possible that TRPM8 may exhibit some voltage-sensitive activity [44]. If this exists, a membrane voltage change resulting from modulation of other voltage-dependent channel activity, such as voltage-dependent sodium channels that are also expressed in RB cells, may in turn affect TRPM8 channel activity (Figure 6) [44,45,46,47]. Yapa et al. suggested that some effects of the TRPM8 antagonist, AMTB, may stem from an interaction with other membrane receptors. Our results shown in Figure 5a are consistent with the notion that the effect of AMTB includes suppression of voltage changes that activate TRPM8 [35]. Another possibility for TRPM8 activation in tumor cells could be via the body’s own hormones, as has already been observed in uveal melanomas in connection with vascular endothelial growth factor (VEGF) and TRPM8 (thyronamine) [48]. Thyronamines, such as 3-T_1_AM, can specifically activate TRPM8 [49,50] (Figure 6). Therefore, the mechanisms of interactions between TRPM8 and CB1 (CB1-TRPM8 axis) are complex.

A noteworthy finding of this study is that NGF and WIN55,212-2 pretreatment solely blocked subsequent TRPM8 activation induced by icilin in the etoposide-resistant WERI-ETOR cells (Figure 4 and Figure 5b, pink bar). Accordingly, we compared the effects of changing the order of presentation of NGF and WIN55,212-2 on icilin-induced activation of TRPM8 in two different groups (Figure 5). On the one hand, the effect was evaluated of first applying NGF (Figure 5a) (NGF-group) with that induced by icilin in the two different cell types (Figure 5b) (icilin group).

In the icilin-group, the results shown in Figure 4 indicate that there are differences between WERI-Rb1 and WERI-ETOR cells. On the other hand, applying NGF first (Figure 5a) resulted in a larger Ca^2+^ influx in WERI-ETOR (Figure 5a, green bars), in the presence of WIN55,212-2 (CB1 activation) in WERI-Rb1. This difference is also apparent in the WERI-ETOR cells, but at a significantly lower level (Figure 5a, red bars). One explanation for this difference could be that NGF triggers a suppression of intracellular Ca^2+^ when CB1 is activated only in WERI-ETOR cells since there is no difference in the WIN55,212-2-induced Ca^2+^ influx between WERI-Rb1 and WERI-ETOR cells (Figure S1). On the other hand, treatment with NGF alone led to more dead cells in WERI-Rb1 cells compared to that of WERI-ETOR cells, which are more resilient (Figure 2e). Interestingly, the NGF-induced Ca^2+^ increase was at higher levels in WERI-ETOR without WIN55,212-2 pretreatment (Figure 5a, green bars). This larger Ca^2+^ influx is due to plasma membrane TRPM8 channel activation, since NGF does not influence Ca^2+^ regulation in Ca^2+^ free conditions in both WERI-Rb1 and WERI-ETOR cells (Figure S2).

It is conceivable that both the CB1 functional activity and its gene expression level affect its crosstalk with TRPM8 and NGF. Such communication modulates Ca^2+^ influx by acting as a possible fine-tuning switch that suppresses the Ca^2+^ overload and, thus, cell survival. Possibly, such modulation may also affect the development of the etoposide resistance in WERI-ETOR cells. Genetically, *CB1* mRNA expression levels in NGF-treated WERI-ETOR cells are clearly upregulated (Figure 1k). A higher CB1 receptor density may, therefore, downregulate Ca^2+^ influx via TRPM8 in the presence of NGF and WIN55,212-2 in WERI-ETOR cells. The identity of possible NGF-induced Ca^2+^ signal transduction pathways that trigger via CB1 stimulation increases in TRPM8 activity may be more complex, as shown in the model in Figure 6. Overall, the lower Ca^2+^ levels in WERI-ETOR cells are only observed when NGF is applied in combination with WIN55,212-2 by WIN55,212-2 or by applying both WIN55,212-2 and icilin (Figure 4). Accordingly, this may potentially endow WERI-ETOR cells with a greater chance of survival than normal WERI-Rb1 cells since a possible Ca^2+^ overload may induce apoptosis which is possibly suppressed by the described downregulation of Ca^2+^ channels [42]. A deeper understanding of the signaling pathways constituting the GPCR-TRP axis [20], such as that involving NGF interaction with the CB1-TRPM8 axis, may facilitate the development of more selective and effective therapies to trip attainment of Ca^2+^ overload and apoptosis in cytostatic-resistant cells, such as the WERI-ETOR cells.

### 3.5. Limitations of This Study

There are some factors that limit our interpretation of the physiological relevance of this study. On the one hand, some technical limitations reduce the precise calcium imaging measurements. We encountered a relatively large number of variations in both scatter signal–noise ratios, which is evident in the results shown in Figure S2. This variability is mostly due to weak fluorescence signals, which may be due to differences in fura-2/AM dye loading. Unusually, linearly large rises in fluorescence signals were discarded from the analysis, as they may be due to the presence of poorly functional or dying cells in which calcium homeostasis is disrupted. Since the RB cells are floating cultures, adhesion to the coverslip was also limited even when they were well coated with polylysine. The very compact RB cells can also easily slip away from the region of interest, which can lead to measurement inaccuracies. To circumvent this problem, the number of replicates or the number of cells interrogated was increased. The bleaching effects (e.g., due to excessive dyeing) could be alternatively compensated by drift correction using TIDA software V. 5.25 (HEKA Electronic, Lamprecht/Pfalz, Germany).

In addition to the technical limitations, it is necessary to bear in mind that data extrapolation from the in vitro to the in vivo condition may be compromised. On the other hand, the WERI-Rbl cell line exhibited a phenotype that is consistent with malignant cells. Namely, they are capable of indefinite growth, tumorigenicity, and the tendency to deviate from the normal diploid karyotype [54]. In addition, neuroblastic differentiation potential of WERI-Rbl cells may not be maintained in an organ culture system [55]. Another limitation involves usage of icilin as a TRPM8 agonist. Although it could be shown in a study by McKemy about cold- and menthol-sensitive receptors (CMR1) that icilin possesses about 2.5-fold greater efficacy and nearly 200-fold more potency than menthol [56], it can also stimulate TRPA1 and the (delta subunit of) human epithelial sodium channel [57,58]. Nevertheless, icilin was frequently used as a relatively selective TRPM8 agonist in other types of tumor cells [13,48].

### 3.6. Clinical Relevance and Outlook

Many diverse mechanisms have been proposed to account for how tumor cells develop drug resistance to certain cytostatic drugs. Such differences are ascribable to tumor cell phenotypic heterogeneity. The model based on the current study is consistent with another study in which it was proposed that cannabinoid receptors and TRPs cooperate in controlling the development of multidrug resistance [59]. Despite such insight, further research is needed to better delineate improved therapeutic management procedures of cancer. This endeavor depends on implementing the usage of small molecules [60,61,62] or monoclonal antibodies [63,64,65], to improve both the therapy and management of cytostatic drug-resistant tumor cells.

### 3.7. Conclusions

This study describes interactions between NGF and CB1 that modulate control of TRPM8 activation and, in turn, regulate etoposide sensitivity in RB cells. Differences were probed between WERI-Rb1 etoposide-sensitive tumor cells and their etoposide-resistant WERI-ETOR counterparts. In the presence of NGF and WIN55,212-2, icilin only failed to induce TRPM8 activation in etoposide-resistant WERI-ETOR cells (Figure 4 and Figure 5b). This difference suggests that there is an association between changes in TRPM8 expression levels and etoposide sensitivity. Nevertheless, the involvement of TRPM8 in mediating the control of etoposide sensitivity is still unclear. This uncertainty exists because a disconnect exists between the magnitudes of NGF-induced rises in Ca^2+^ influx in WERI-ETOR cells and the larger cytotoxicity of etoposide in WERI-Rb1 cells. From the literature, it is generally known that calcium overload is toxic for the cell and that it is associated with an increase in apoptosis [43] and, thus, a poorer cell survival. Although large WIN55,212-2-induced Ca^2+^ influxes were observed in both cell lines, the Ca^2+^ influxes were at lower levels in the presence of NGF only in WERI-ETOR cells (Figure 5a, red bars). This was not the case in WERI-Rb1 cells, in which a higher probability of Ca^2+^ overload above a certain threshold increases the probability of inducing apoptosis.

## 4. Materials and Methods

### 4.1. Materials

Cell culture medium and other cell culture supplements were purchased from Biochrom AG (Berlin, Germany) or GIBCO Invitrogen (Karlsruhe, Germany). All reagents (e.g., trypan blue T8154) were purchased from Sigma-Aldrich (Diesenhofen, Germany) unless otherwise specified. Recombinant Human β-NGF was purchased from Biogems company (Westlake Village, CA, USA). Icilin, AM251, and WIN55,212-2 were purchased from Cayman Chemical Company (Ann Arbor, MI, USA). For calcium imaging, the fluorescent dye fura-2/AM was purchased from PromoCell GmbH (Heidelberg, Germany).

### 4.2. Cell Culture

Etoposide-sensitive and -resistant WERI-Rb1 cell lines were used for the measurements. The cell lines were kindly provided by Stephan et al. (Essen, Germany). Furthermore, an established cultivation was carried out. Both cell lines were cultured in RPMI-1640 medium supplemented with 10% fetal bovine serum (FBS), and 100 IU/mL penicillin/streptomycin was added for antibacterial protection. Cells were cultured in an incubator at 37 °C, 80% humidity, and 5% CO_2_. For the measurements, the cells were seeded on 12 multiple cell culture plates. Cells were coated with Pol-L-lysine on the coverslips and fixed accordingly for measurements. All measurements were performed in a period of 48–72 h after preparation. Medium exchange took place in the cell culture three times per week.

### 4.3. Cell Viabilty

For each cell line, a control experiment was performed without the drug. The experiment was scheduled for five days. Then, 100 ng/mL NGF was added to the cells on the first day to check for a possible effect of NGF on cell proliferation or apoptosis. On the third day, 1 mL of fresh RPMI medium was added to the cells. On the fifth day, the proliferation and apoptosis measurements were performed. For more accurate counting of dead cells, 0.4% trypan blue dye was used. A Neubauer counting chamber (A. Hartenstein GmbH, Würzburg, Germany) was used to perform the measurements to calculate the live and dead cells.

### 4.4. mRNA Purification, cDNA Synthesis, and RT-qPCR Analyses

For mRNA isolation, etoposide-sensitive WERI-Rb1 cells and etoposide-resistant WERI-ETOR cells were cultured and frozen in liquid nitrogen as described previously [9,15]. Total RNA extraction was conducted using the Gene Elute Mammalian Total RNA Miniprep Kit (Sigma-Aldrich, St. Louis, MO, USA) following the manufacturer’s guidelines. The purity and concentration of the RNA were assessed using a BioSpectrometer (Eppendorf, Hamburg, Germany).

For cDNA synthesis, 1 µg of RNA was reverse-transcribed utilizing the First Strand cDNA Synthesis Kit with random hexamer primers (Thermo Fisher Scientific, Breda, The Netherlands).

RT-qPCR analyses were conducted utilizing the FastStart Essential DNA Green Master Mix in a Light Cycler 96 instrument (Roche Applied Science, Mannheim, Germany). The reaction conditions were as follows. First, an initial pre-incubation step at 95 °C for 10 min was carried out, followed by 45 cycles of amplification with the following parameters: 10 s at 95 °C, 30 s at 60 °C, and 10 s at 72 °C. Additionally, melting curve analyses were performed with the following settings: 10 s at 95 °C, 60 s at 65 °C, and 1 s at 97 °C. Finally, a cooling step at 37 °C for 30 s was included. Primer pairs for RT-qPCR were designed using the ProbeFinder Assay Design Center (Roche Applied Science; see Table 1). The primer efficiency of each primer pair was determined based on a cDNA dilution series ranging from 5 ng to 125 ng. The RT-qPCR data are expressed as the median ± quartile range, with the maximum and minimum values included. Statistical analysis was performed using the pairwise fixed reallocation and randomization test (REST 2009 released Qiagen, [66]).

### 4.5. Fluorescence Calcium Imaging

WERI-Rb1 cells grown on coverslips were preincubated with 1 µM fura-2/AM (1 mM stock solution) at 37 °C for 20–40 min. After the incubation period, coverslips containing the cells were thoroughly rinsed with a Ringer-like (control) solution (RLS) containing (in mM): 150 NaCl, 6 CsCl, 1 MgCl_2_, 1.5 CaCl_2_, 10 D-glucose, and 10 HEPES (pH ≈ 7.4; osmolarity ≈ 316 mosmol/L). Coverslips were placed into a bath chamber and immediately rinsed with 1.5 mL of RLS under a fluorescence microscope (Olympus BX50WI; Olympus Europa Holding GmbH, 20097 Hamburg, Germany) equipped with a Omikron V. 1.0 software-controlled high-powered fluorescence LED light source (LED-Hub by Omikron, Rodgau–Dudenhoven, Germany), a high-resolution digital camera (Olympus XM10), and a peristaltic pump P-1 (Pharmacia, London, UK) connected to the bath chamber in a dark room at room temperature (RT) (~23 °C). Single cells were selected and designated as regions of interest (ROIs) using the cellSens Dimension V. 1.16 software, and excited by alternating illumination at 340 and 380 nm in 5 s intervals through a software-controlled interface (Olympus U-RTC, cellSens Dimension V. 1.16). Fluorescence fura-2 emission was recorded at 510 nm (120 frames per experiment for a total experimental time of 10 min). The fluorescence ratio f340 nm/f380 nm of the two wavelength-traces correlates directly with the changes in intracellular Ca^2+^ level ([Ca^2+^]_i_) as described by Grynkiewicz et al. [67]. Fluorescence ratios were calculated with cellSens software and normalized (control set to 0.1), drift corrected (if applicable), and averaged (with SEM error bars) using the TIDA V. 5.25 software for Windows (HEKA Electronic, Lamprecht/Pfalz, Germany). The results are depicted as mean traces of f340 nm/f380 nm ratio ± SEM with *n*-values indicative of the number of measured cells per data point. Drug effects were evaluated following a ~30 min pre-incubation time. Drug stock solutions were prepared with DMSO and diluted in RLS so that the final DMSO concentration was below 0.1%. At this concentration, it was not per se cytotoxic for ocular cells [68,69].

### 4.6. Statistical Data Analyses

Paired data were probed for normality according to the Kolmogorov–Smirnov test, and the Student’s *t*-test assessed statistical significance of paired data if they passed normality. Alternatively, the Wilcoxon matched pairs test was instead used if they failed normality. Likewise, statistical significance was determined for unpaired data using Student´s *t*-test if they passed normality or by Mann–Whitney U test if they failed normality testing. Probabilities of *p* < 0.05 (indicated by asterisks for paired data (*) and hash tags (#) for unpaired data) were considered significant. Statistical tests were performed, and diagrams were created using SigmaPlot version 12.5 for Windows (Systat Software, Inc., Point Richmond, CA, USA) as well as GraphPad Prism software version 5.00 for Windows (La Jolla, CA, USA). The number of replicates is shown in each case in brackets, near the traces or bars. All values are given as means ± standard error of the mean (SEM) (error bars in both directions).

## Figures and Tables

**Table 1:** Primer pairs used for RT-qPCR analyses. To assess the mRNA expression levels of *CNR1*, *NGF*, and *TRPM8*, the expression of the housekeeping genes *β*-*Actin* (*ACTB*) and *S18* (*RBPS18*) was analyzed for normalization and relative quantification. Each gene’s primer sequence and the expected amplicon size are provided. Abbreviations: bp = base pairs, for = forward, rev = reverse.

Primer	Sequence	Product Size (bp)	GenBank Accession Number/ Reference
*ACTB* For	CCAACCGCGAGAAGATGA	97	NM_001101.3; [15]
*ACTB* Rev	CCAGAGGCGTACAGGGATAG		
*CNR1* For	CTGCAGAGCTCTCCGTAGTC	125	NM_016083.6; this study
*CNR1* Rev	GGGGGCAATCCCTTCGC		
*NGF* For	GAGCGCAGCGAGTTTTGG	127	NM_002506.3; this study
*NGF* Rev	TGGCCAGGATAGAAAGCTGC		
*RPS18* For	CTTCCACAGGAGGCCTACAC	82	NM_022551.2; [9,15]
*RPS18* Rev	CGCAAAATATGCTGGAACTTT		
*TRPM8* For	GGTCCTGTACTCGCTGGTCT	67	NM_024080; [9]
*TRPM8* Rev	CACCCCATTTACGTACCACTG		

## Data Availability

The data presented in this study are available on request from the corresponding author. The data are not publicly available due to privacy limitations.

## Supplementary Materials

The following supporting information can be downloaded at: https://www.mdpi.com/article/10.3390/ijms25031733/s1.
