# Supplementary material for: Joint CB1 and NGF Receptor Activation Suppresses TRPM8 Activation in Etoposide-Resistant Retinoblastoma Cells
Source: Int J Mol Sci. 2024 Jan 31;25(3):1733. doi: 10.3390/ijms25031733 (PMC10855132; doi:10.3390/ijms25031733)
Supplement: Supplementary file 1 [file ijms-25-01733-s001.zip › ijms-2813575-supplementary.pdf]

Supplementary Materials

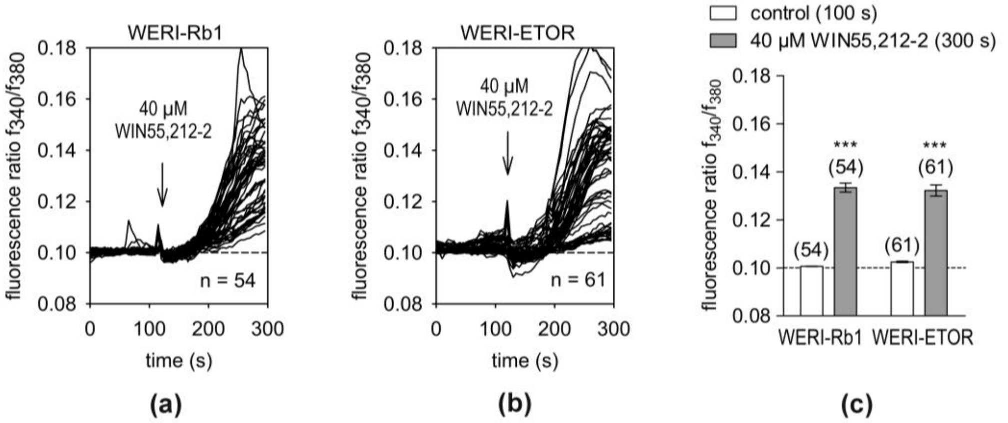

Figure S1: WIN55,212-2 increases intracellular Ca2+ in WERI-Rb1 and WERI-ETOR cells at the same level. (a) The time dependent changes are shown as relative intracellular Ca2+ levels in fura2-loaded WERI-Rb1 cells. Data are represented in single traces. n indicates the number of cells examined in this set of experiments (n = 54). The dashed line represents the reference line for baseline value (0.1). The arrow indicates the point of time of application of WIN55,212-2 (40 µM). (b) Same experiment as shown in panel (a) but with WERI-ETOR cells (n = 61). Extracellular application WIN55,212-2 induced an increase in Ca 2+ influxes, which are comparable with those of WERI-Rb1 cells. (c) Summary of the experiments with WIN55,212-2. Columns represent mean values ± SEM of fluo-rescence ratio at 100 and 300 s. The numbers of cells measured in both experiments are indicated in brackets above the columns. The asterisks (***) designate significant increases in [Ca2+]i in the presence of WIN55,212-2 (gray columns at t = 300 s; n = 54 - 61; ***p < 0.001; paired tested).

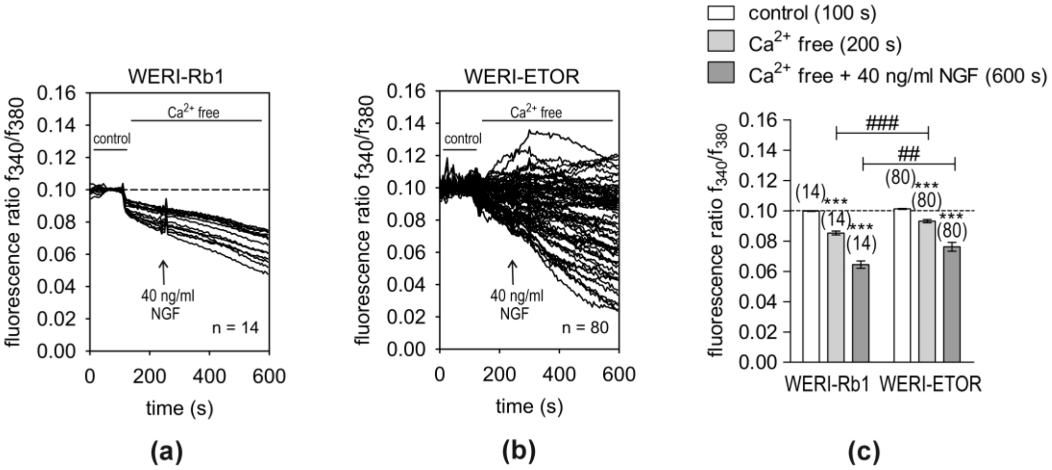

Figure S2: NGF does not change intracellular Ca2+ in WERI-Rb1 and WERI-ETOR cells in Ca2+ free conditions. (a) The time dependent changes are shown as relative intracellular Ca2+ levels in fura2-loaded WERI-Rb1 cells. Data are represented in single traces. n indicates the number of cells examined in this set of experiment (n = 14). The dashed line represents the reference line for baseline value (0.1). The arrow indicates the point of time of application of NGF (40 ng/ml). (b) Same ex-periment as shown in panel (a) but with WERI-ETOR cells (n = 80). Extracellular application NGF induced a heterogeneous increase in Ca 2+ influxes, in comparison with the WERI-Rb1 cells. (c) Summary of the experiments with NGF experiment in Ca2+ free conditions. Columns represent mean values ± SEM of fluorescence ratio at 100, 200 and 600 s. The numbers of cells measured in both experiments are indicated in brackets above the columns. The asterisks (***) designate signif-icant

decreases in [Ca2+]i in the presence of a Ca2+ free RLS (gray columns at t = 200 and 600 s; n = 14 – 80; ***p < 0.001; paired tested). The hashtags (##) designate significant differences in the decreases in [Ca2+]i between WERI-Rb1 and WERI-ETOR (gray columns at t = 200 and 600 s; n = 14 – 80; ##p < 0.01 at the minimum; unpaired tested).
